# Oncofoetal insulin receptor isoform A marks the tumour endothelium; an underestimated pathway during tumour angiogenesis and angiostatic treatment

**DOI:** 10.1038/s41416-018-0347-8

**Published:** 2018-12-18

**Authors:** Patrycja Nowak-Sliwinska, Judy R. van Beijnum, Elisabeth J. M. Huijbers, Paula C. Gasull, Laurie Mans, Axel Bex, Arjan W. Griffioen

**Affiliations:** 1 School of Pharmaceutical Sciences, University of Geneva and University of Lausanne, Geneva, Switzerland; 2 Translational Research Center in Oncohematology, University of Geneva, Geneva, Switzerland; 3 Angiogenesis Laboratory, Department of Medical Oncology, Amsterdam UMC, Vrije Universiteit Amsterdam, Cancer Center Amsterdam, Amsterdam, The Netherlands; 4 Department of Urology, The Netherlands Cancer Institute, Amsterdam, The Netherlands

**Keywords:** Tumour angiogenesis, Targeted therapies

## Abstract

**Background:**

In a genomic screen for determinants of the tumour vasculature, we identified insulin receptor (INSR) to mark the tumour endothelium. As a functional role for insulin/INSR in cancer has been suggested and markers of the tumour endothelium may be attractive therapeutic targets, we investigated the role of INSR in angiogenesis.

**Methods:**

In a genomic screen for determinants of the tumour vasculature we identified insulin receptor to mark the tumour endothelium.

**Results:**

The current report demonstrates the following: (i) the heavy overexpression of INSR on angiogenic vasculature in human tumours and the correlation to short survival, (ii) that INSR expression in the tumour vasculature is mainly representing the short oncofoetal and non-metabolic isoform INSR-A, (iii) the angiogenic activity of insulin on endothelial cells (EC) in vitro and in vivo, (iv) suppression of proliferation and sprouting of EC in vitro after antibody targeting or siRNA knockdown, and (v) inhibition of in vivo angiogenesis in the chicken chorioallantoic membrane (CAM) by anti-INSR antibodies. We additionally show, using preclinical mouse as well as patient data, that treatment with the inhibitor sunitinib significantly reduces the expression of INSR-A.

**Conclusions:**

The current study underscores the oncogenic impact of INSR and suggests that targeting the INSR-A isoform should be considered in therapeutic settings.

## Background

Angiogenesis is an intricately regulated process required during normal development and a large array of different pathologies. Strategies to inhibit blood vessel formation are therefore considered a promising therapeutic approach.^1,2^ Although the introduction of anti-angiogenic therapies in the clinic has shown a clear improvement of cancer therapy, the benefit for patient survival is still rather limited.^3^ Induction of drug resistance seems to be a major cause of this limited efficacy.^4,5^ Targeting the vasculature directly, independent of tumour produced growth factors, seems to be beneficial in this regard, also because of the fact that endothelial cells (EC), even when associated to the tumour, are genetically stable and do not easily mutate to resist treatment. Previously identified tumour EC targets^6,7^ were most often neither fully specific for the tumour endothelium, nor were they absent in physiologically activated endothelium, rendering them inferior for anticancer strategies in patients. Therefore, the identification of unique targeting molecules on tumour EC is urgently needed.

We have previously identified several markers of angiogenic tumour EC.^6,8,9^ Using cDNA libraries from freshly isolated colorectal tumour EC, patient-matched normal colon tissue EC and placenta derived EC, a broad profile of differentially expressed genes in tumour endothelium, but not physiologically activated placenta EC, was generated.^9^ Furthermore, we have shown that targeting these markers can have therapeutic effects in animal models.^9–13^

In this study, we demonstrate that the insulin receptor (INSR) is one of these tumour EC markers having a clear role in tumour angiogenesis and a potential impact for the clinical management of cancer therapy. The insulin signalling axis consists of, next to insulin, two structurally related ligands, i.e. insulin-like growth factor (IGF)-1 and -2, a set of receptors, i.e. INSR, IGF1R and IGF2R and a family of seven insulin-like growth factor-binding proteins (IGFBP).^14^ A large body of research has been performed on IGF1R and its function in cancer is well documented.^14,15^ Anti-IGF1R strategies have been clinically used to treat cancer. The role of INSR in cancer is less well studied but a role for insulin and INSR has been suggested.^16,17^ Moreover, associations between insulin treatment and the occurrence of cancer have been observed.^18,19^ A role for INSR in cancer therapy is becoming eminent since it has been suggested that the non-metabolic, oncofoetal isoform INSR-A is the variant of INSR that is overexpressed in cancer.^20,21^ Targeting only INSR-A would then become possible without intervention in the metabolic functions of the insulin/INSR pathway. Since INSR-A is a shorter isoform, lacking exon 11, which is present in the full-length protein INSR-B, it might be difficult but not impossible to specifically target INSR-A.

The current study describes the overexpression of INSR, which is mainly INSR-A, in the vasculature in a variety of human cancer types, where it is observed to predict decreased patient survival. Since mice are metabolically insensitive to insulin overstimulation and deprivation, extensive mechanistic investigation of specific targeting of INSR-A in a mouse model is not feasible. We present here for the first time the finding on the embryonic origin of the INSR-A isoform and its overexpression in the tumour vasculature. Our results on intervention in the insulin/INSR signalling axis by siRNA and antibody-mediated targeting in vitro and in vivo demonstrate the capacity of this pathway to regulate tumour angiogenesis. The current insights suggest that an INSR-A targeting strategy may be an attractive angiostatic cancer treatment.

## Materials and methods

### Isolation of endothelial cells and tissue processing for transcriptome analysis

Endothelial cells were immuno-isolated from freshly resected colorectal tumours and patient-matched normal colon as described previously.^9,22^ Similar procedures were applied to isolate EC from murine tumours (Supplementary data). For the comparison of EC fractions with whole organism profiles, mouse embryos and adult mice were used (Supplementary data). All RNA was isolated according to the TRIzol reagent (Life Technologies) protocol.

### RNA sequencing

Next generation sequencing was performed to analyse the transcriptomes of sorted mouse tumour endothelial cells (TEC), whole embryos and adult mice to profile re-expression of embryonic genes in TEC. PolyA ^+^ RNA selection was performed to select for stable mRNA and to deplete small RNAs, tRNA and bacterial or other prokaryotic RNA. For each sample a sequencing library was prepared with 50 base paired end reads (Illumina TruSeq Sample Prep protocol) to enable detection of alternative splicing. Samples were sequenced on an Illumina HiSeq 2000 (Illumina, San Diego, CA) with HCS 2.2.68 software suite (Illumina). RNA sequencing experiments and analysis of the data was performed by the genomics core facility at the Netherlands Cancer Institute. The obtained reads (50 million 50 bp paired end reads per sample) were mapped to the mouse reference genome (GRCm38). Genome mapping and differential expression analysis was performed with Cufflinks software.^23^ For detection of splice variants, the reads were mapped to the whole mouse genome, including exon and intron DNA.

### Immunohistochemical staining

Immunohistochemical staining of INSR in paraffin sections of human tumours and corresponding normal tissues was performed using mouse monoclonal anti-INSR antibody (AS53586, Tebu-Bio; 1:100) according to previously described protocols.^24,25^ Quantification was performed by blinded evaluation of coded samples by two independent observers, following a score ranging from lack of expression (score 0) to very high expression (score 4). The expression was quantified in 11 different tumour types with up to 20 samples per tumour type of both in-house stained tissues and tissue staining from the human protein atlas (www.proteinatlas.org).

### Kaplan–Meier curves

Kaplan–Meier scanner functionality in R2 (R2: Genomics Analysis and Visualization Platform; http://r2.amc.nl) was employed on three available colorectal cancer Affymetrix gene expression data sets^26,27^ using average INSR gene expression as selection parameter. Relapse-free survival at 120 months was taken as evaluation point.

### Cell viability and proliferation assay

Endothelial cells (5 × 10^3^ cells/well for HUVEC, 1 × 10^4^ cells per well for HMEC) were seeded in gelatine-coated 96-well cell culture plates as described previously.^10^ Briefly, 24 h after seeding, culture medium with or without compounds was added and cells were grown for an additional 72 h. Cell viability was assessed using the CellTiter-Glo^®^ luminescent Cell Viability Assay (Promega, Madison, WI) according to the manufacturer’s instructions. Tritium-thymidine incorporation to measure cell proliferation by DNA synthesis was performed as previously described.^28^

### Endothelial cell migration assay and sprouting assay

Endothelial cell migration was performed using a guided 96-well pin tool (Peira, Turnhout, Belgium) and Leica DMI3000 microscope (Leica, Rijswijk, The Netherlands) with Universal Grab 6.3 software (DCILabs, Keerbergen, Belgium), at time points *T* = 0 h and *T* = 6 h.^29^ Wound closure (μm^2^) was expressed as a percentage of control wells. EC spheroids were created using the hanging drop technology,^30^ as detailed in the Supplementary data, and quantification of sprouting was performed using a semi-automatic Image-J-based macro.^31^

### Patient tissues

Primary tumours from patients with RCC treated prior to surgery with sutent (*N* = 21) were used for the evaluation as described previously.^24^ The tissues of 12 primary tumours came from EudraCT 2006-006491-38 (https://eudract.ema.europa.eu/) phase II trial where the main objective was the investigation of the response rate of the primary RCC tumour to sunitinib at 50 mg/day for 2 cycles of each 4 weeks on treatment followed by 2 weeks off treatment. At completion of the 2^nd^ cycle patients underwent cytoreductive nephrectomy as per protocol 1 day after discontinuation of sunitinib. Remaining 9 primary tumours were provided from a second phase II study (NCT00715442) of prior to surgery treatment with sutent in patients with primary clear cell mRCC. These patients were restaged after one cycle of systemic therapy, began a second cycle of systemic therapy with sunitinib, and discontinued therapy 1 day before nephrectomy. Clear cell RCC tissues from non-treated patients were used as controls.

### Chorioallantoic membrane of the chicken embryo (CAM) assay

Detailed methods on growth, handling and treatments of the eggs are described in the Supplementary Data. Insulin activity in developmental chicken embryo CAM assay^30^ was assessed via topical administration of insulin (on embryo development day 7 and 8) at the indicated concentrations. Vasculature was visualised and analysed on embryo development day 9 as previously described.^29^

Visudyne^®^*-*photodynamic therapy (PDT) was performed^32^ on embryo development day 11. Within PDT-treated areas, 20 μl anti-INSR monoclonal or polyclonal antibodies (10 μg/ml) were administered topically twice, immediately after PDT and 24 h later. Quantification based on the fluorescence angiographies was performed on embryo development day 13.^33^

### Sunitinib treatment

Animal experiments were approved by the local Ethical Review Committee and performed as described previously.^34^ Four- to six-week-old male severe combined immunodeficient (SCID) mice were housed under pathogen-free conditions. Mice were injected subcutaneously with 5 × 10^6^ HT29 cells. Mice received treatment with sunitinib malate (40 mg/kg) or a corresponding amount of vehicle, once daily, 7 days a week, by oral gavage. After 6–8 weeks of treatment, animals were sacrificed; tumours were harvested and subsequently snap-frozen in liquid nitrogen for qPCR.

### Tissue processing and qPCR

Tumour specimens from patients with kidney malignancies (*N* = 5) and their paired healthy kidney tissues (*N* = 5) were also collected and snap-frozen in liquid nitrogen. Visudyne^®^-PDT-treated zones (see below) of the CAM were fixed. Samples were processed for RNA isolation using TRIzol Reagent. Cultured cells and mouse HT29 tumours were subject to RNA isolation using RNeasy mini columns (Qiagen, Venlo, The Netherlands) according to the manufacturers’ instructions. Primers were designed according to previously published guidelines,^35^ to allow detection of species-specific transcripts. This was, however, not possible for the variant-specific detection due to (i) absence of the long (INSR-B homologous) isoform in chicken and (ii) too extensive homology between mouse and human in the region of exon 11.

qPCR was performed using SYBR green reagent (Bio-Rad), in a 2-step protocol at a Tm of 60°C on a CFX96 thermal cycler (Bio-Rad) and runs were analysed using CFX manager software (Bio-Rad). PCR primers are listed in Supplementary Table S1. The relative expression was calculated relative to three genes, i.e. cyclophilin-A (PPIA), Actin-β (ACTB) and beta-2-microglobulin (B2M), using the 2^-dCt method as described previously.^35^

### Statistical analysis

All values are given as mean values ± SEM. Statistical analyses were done using *t-test*, Mann–Whitney U-test or one-way ANOVA in GraphPad Prism. Where relevant, the Bonferroni post-test was used to correct for multiple comparisons. *P* values < 0.05 were considered statistically significant.

## Results

### Insulin receptor (INSR) is a marker of the tumour vasculature

**Fig. 1:**
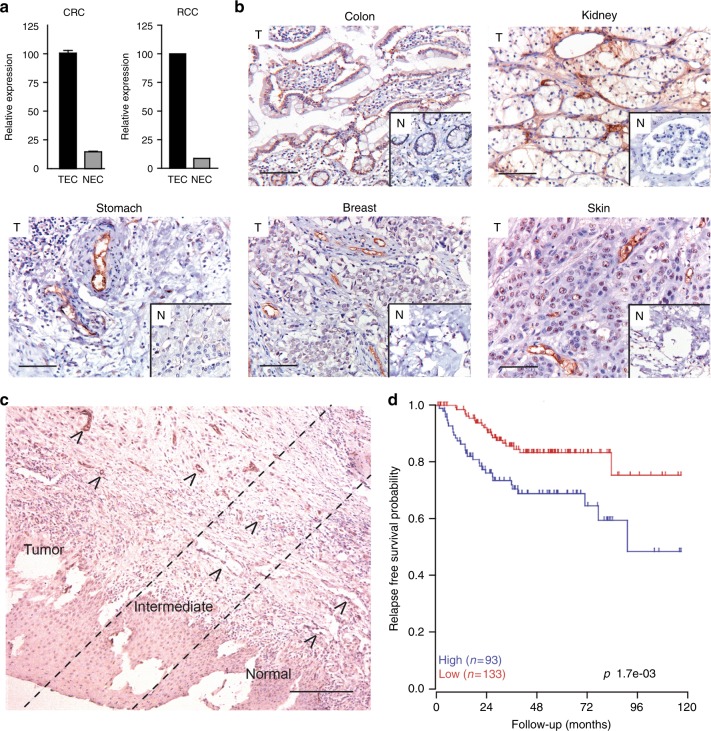
Insulin receptor (INSR) is overexpressed in tumour endothelial cells. **a** Overexpression of INSR in colorectal cancer (qPCR;^9^) and renal cell cancer (proteomics;^55^) associated endothelial cells. **b** Immunohistochemical staining for INSR on sections of different paraffin embedded human solid tumours (colon, kidney, stomach, breast, skin) and normal tissue counterparts. A clear induction of vascular staining (brown) is seen tumours (T) as compared to normal tissues (insets; N). Scale bars represent 50 μm. **c** Immunohistochemical staining for INSR of human head and neck squamous carcinoma tissue with adjacent normal tissue. Overexpression of INSR is related to malignancy as strong vascular staining is observed in the tumour, less pronounced staining in the intermediate area and absence of staining in the adjacent normal tissue. Arrows heads highlight the blood vessels. The scale bar represents 200 μm. **d** Kaplan–Meier survival analysis of 226 CRC cases based on average INSR expression

In a genomic search for specific tumour endothelial markers, we compared the transcriptomes of sorted colon carcinoma tumour endothelial cells (TEC) and normal colon endothelial cells (NEC). Endothelial cells (EC) of human placenta (PLEC) were used for comparison to a source of physiologically activated EC. We identified insulin receptor (INSR) as a specifically and highly overexpressed gene in the tumour vasculature of colorectal carcinoma (CRC) and validated this by qPCR. A similar result was found for EC from renal cell cancer tissue (RCC) (Fig. 1a). Immunohistochemical staining for INSR in CRC tissue confirmed the overexpression in the tumour vasculature, as compared to vessels in normal tissues, where INSR expression was very low or absent. Similar observations were done in kidney-, stomach-, breast- and skin carcinoma tissues (Fig. 1b). Interestingly, in all tumour tissues expression of INSR was rather exclusively observed in the vasculature.

The overexpression of INSR in tumour vessels was further assessed and quantified in a series of 11 different tumour types. A strong to very strong expression was observed in the vasculature of these tumours, while vessels in the corresponding normal tissues showed only negligible expression (Supplementary Fig. S1; Supplementary Fig. S2A). The relationship between malignancy and the overexpression of INSR is best shown in a tissue section representing the rim of a tumour, e.g., of a head and neck squamous cell carcinoma and the adjacent normal tissue. A gradual increase of endothelial INSR expression is seen in the transition from normal to malignant tissue (Fig. 1c). Overexpression on activated EC was further demonstrated in cultured EC by applying conditions of starvation and exposure to growth factors. While starved and slow-growing HUVEC show a low expression of INSR, the molecule is significantly induced by the mitogenic signals under growth factor activated conditions (**p < 0.01, Supplementary Fig. S2B). Relatedly, it was observed that the fast-growing HMEC express higher levels of INSR mRNA than slow-growing HUVEC, independently suggesting a relationship with the growth rate of cells (**p < 0.01, Supplementary Fig. S2C).

### Expression of INSR is correlated to CRC patient survival

Transcriptome analysis of 226 CRC tissues and stratification of patients for higher and lower than average expression of INSR, demonstrated the negative relation with relapse-free survival of patients (Fig. 1d).^26^ As the expression of INSR is mainly present in the vasculature of tumours, it is suggested that high vascular INSR expression contributes to enhanced angiogenesis and increased tumour aggressiveness. Similar relations were found for the known and therapeutically exploited angiogenic growth factor receptors for VEGF, FLT-1 and KDR (Supplementary Fig. S3).

### Insulin receptor-A (INSR-A) is the main splice variant present in tumour vasculature

**Fig. 2:**
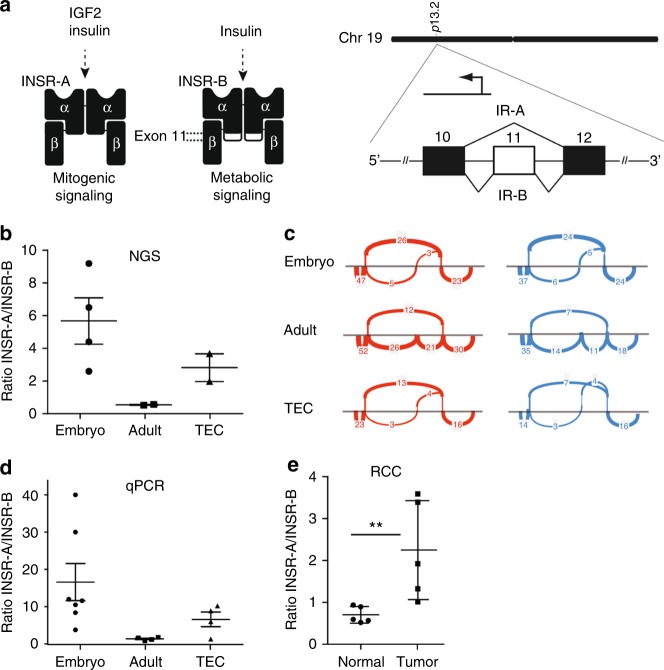
Insulin receptor variant A (INSR-A) is the main splice variant present in tumour endothelial cells. **a** Schematic representation of the two splice variants of INSR, INSR-A (lacking exon 11) and INSR-B (full-length protein). The isoforms differ in ligand affinity and cellular downstream signalling.^14^ **b**, **c** RNA sequencing and variant analysis show the relative ratio of INSR-A over INSR-B in isolated tumour endothelial cells (TEC), mouse embryo and adult mouse, and point to a dominant role for INSR-A in TEC and embryo compared to the adult mouse (ratio INSR-A/INSR-B »1). This is visualised in sashimi plots (**c**), showing that in TEC and embryo the preferential isoform is INSR-A in which exon 11 is omitted. The main variant present in the adult mouse is INSR-B, in which exon 11 is included. **d** Expression of the different splice variants analysed by qPCR confirms these observations. **e** Ratio of INSR-A to INSR-B expression in human renal cell carcinoma (RCC) and healthy kidney tissues, determined by qPCR. **P < 0.01 by Mann–Whitney test, *N* = 5

Alternative splicing of the insulin receptor gene gives rise to two different mRNAs, encoding for the variants INSR-A and INSR-B. The isoform B is the full-length protein that is involved in the metabolic function of INSR, while the mitogenic isoform A represents the shorter variant lacking exon 11 (Fig. 2a). In a search for embryo-specific genes that are overexpressed in tumour endothelial cells, we performed deep RNA sequencing of whole embryo and -adult mouse tissues, as well as sorted tumour endothelial cells (TEC). This analysis revealed INSR-A as one of the embryo-specific markers and it was found to be overexpressed in the tumour vasculature (Fig. 2b), hence, referring to it as an oncofoetal determinant. We used the sashimi plots in order to quantitatively visualise the splice junctions of the different samples. The sashimi plots indicate that mitogenic INSR-A isoform is the main isoform present in TEC, as well as in the embryonic tissue, whereas in the adult mouse tissues INSR-B is the preferential isoform (Fig. 2c and Supplementary Fig. S4). Expression data were confirmed by qPCR (Fig. 2d). We also quantified the expression of INSR isoforms in renal cell carcinoma (RCC) and normal renal tissue. In normal renal tissue the main isoform present is INSR-B, whereas the balance is shifted towards INSR-A in RCC (Fig. 2e).

### Treatment of patients with sunitinib represses INSR in primary renal cell carcinoma tissue

**Fig. 3:**
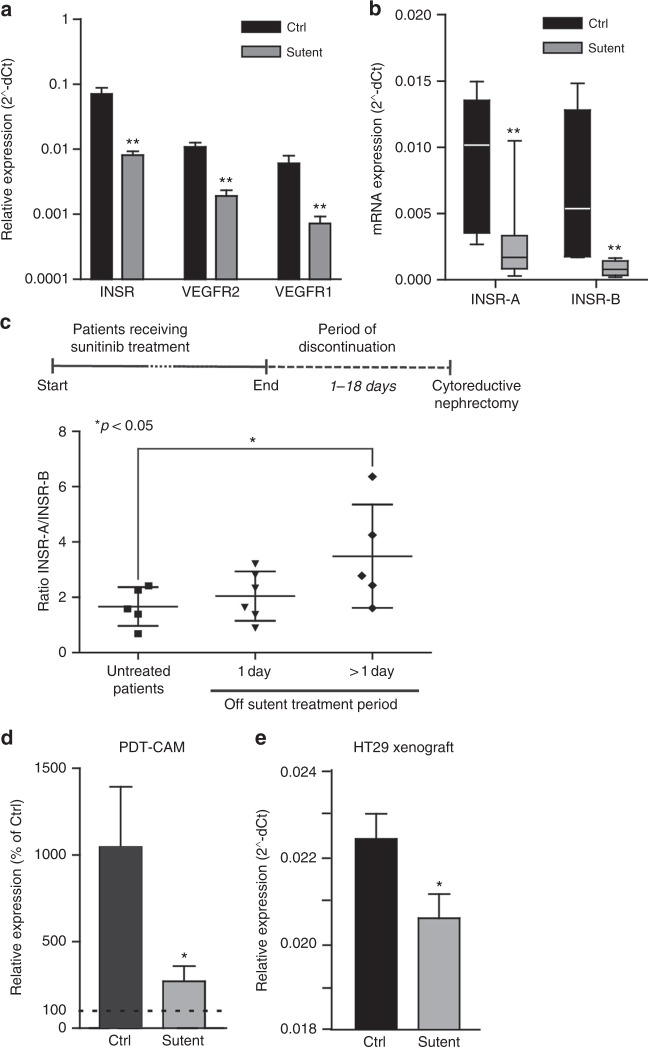
Altered INSR expression after angiostatic treatment with sunitinib. **a**, **b** INSR, VEGFR1 and VEGFR2 expression analysis in human RCC after treatment with sunitinib indicates a reduction expression of these growth factor receptors (A). Both INSR-A and INSR-B show reduced expression (**b**) **P < 0.01 by *t* test, *N* = 8–12, Ctrl vs. sunitinib. (**c**) Ratios of INSR-A vs. INSR-B expression in RCC. Surgery was performed in naive (untreated) patients, or after 1 or more (6–18) days of recovery from 2 cycles of sunitinib treatment prior to surgery. Prolonged discontinuation of sunitinib therapy results in induction of relative INSR-A levels, suggestive of rebound angiogenesis. *P < 0.05 by Mann–Whitney, *N* = 5. **d** qPCR profiling of the CAMs treated with PDT. Results are expressed as % of expression in untreated CAMs. The control bar (Ctrl) represents the increase in INSR expression by the PDT treatment, while the bar labelled sutent represents the normalisation of expression by sutent (20 μg/kg). Both bars present values of the situation 48 h post-PDT. *P < 0.05 (by *t* test). **e** qPCR analysis of HT29 CRC xenografts in mice treated with sunitinib,^34^ indicate that angiostatic treatment reduces vascular INSR expression. Special specific mouse primers identified INSR in mouse stromal tissue exclusively

Sunitinib is the first line treatment for renal cell carcinoma (RCC) patients. In a recent phase II clinical study to investigate the need for removal of the primary tumour in patients with metastasised disease, surgical debulking was performed after two cycles of sunitinib treatment. We previously published that these two cycles of sunitinib treatment effectively inhibited angiogenesis and enhanced the number of mature blood vessels.^24^ In the current study, RNA was isolated from a number of these tumour samples and expression levels of different genes were analysed by qPCR. While INSR is steeply upregulated in tumours as compared to normal kidney (Fig. 1), two cycles of treatment with sunitinib significantly repressed the expression of INSR by up to 8-fold (Fig. 3a). In these tissues a similar significant regulation was found for the VEGFRs^24^ (Fig. 3a). This suppressed expression was observed for both INSR-A and INSR-B (Fig. 3b). As we have previously shown, discontinuation of sunitinib treatment results in a rapid angiogenic rebound resulting in enhanced angiogenesis. Expectedly, extended discontinuation of sunitinib treatment was found to significantly increase the INSR-A/INSR-B ratio (Fig. 3c). Suppression of INSR-B after sunitinib treatment seemed durable (Supplementary Fig. S5B, right), whereas the suppression of INSR-A expression was lost following discontinuation of sunitinib treatment for more than 1 day (Supplementary Fig. S5B left), resulting in the increased ratio of INSR-A over INSR-B and suggesting initiation of rebound angiogenesis (Fig. 3c).

### INSR is suppressed after angiostatic treatment with sunitinib in vivo

It was demonstrated above that INSR expression is upregulated during angiogenic stimulation and inhibited in RCC patients upon anti-angiogenic treatment. To further investigate INSR expression after angiostatic treatment, the CAM experiment of PDT-induced angiogenesis was performed with or without subsequent treatment with the tyrosine kinase inhibitor sunitinib.^36^ While the induced angiogenesis response by PDT resulted in an approximately 10-fold stimulation of INSR expression (dotted line indicates untreated CAM, Fig. 3d), INSR expression was suppressed by sunitinib by approximately 80% to an almost normalised expression. Suppression of vascular INSR after angiostatic treatment was further confirmed in the mouse model of sunitinib-treated HT29 colorectal xenograft carcinomas grown subcutaneously in nude mice (Fig. 3d).^34^ Here, primers were used that selectively amplify mouse INSR transcripts in a background of human cDNA (Supplementary Table S1). However, in these experiments, only total INSR expression was addressed since (i) chicken expresses only the shorter INSR isoform resembling human and murine INSR-A, and (ii) due to the high homology between human and mouse INSR in the region around exon 11, it proved impossible to design valid primers for selective amplification of the variants of either species in a mixture of human and mouse templates.

### Insulin provides an activation signal in endothelial cells in vivo and in vitro

**Fig. 4:**
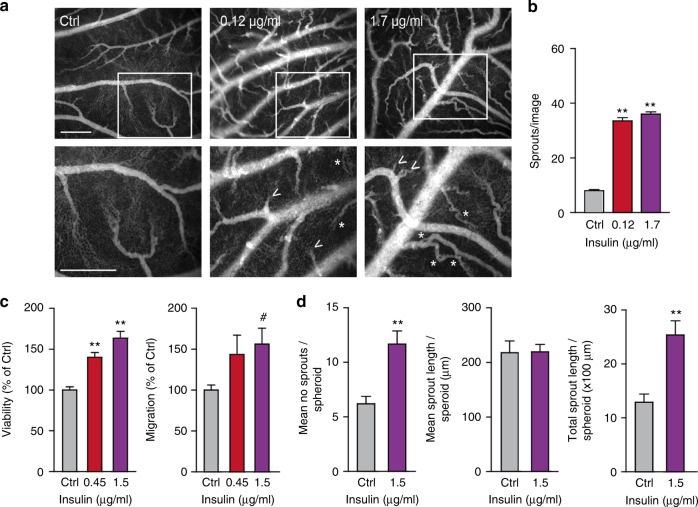
Insulin activates in vivo angiogenesis and in vitro endothelial cell functions. **a** Activation of angiogenesis in vivo in the chorioallantoic membrane (CAM) model. Representative fluorescence angiographies of the CAMs treated topically with various doses of insulin. CAMs were treated topically on development day 7 and 8 (20 μl in 0.9% NaCl) and visualised after i.v. injection of FITC-dextran (10 μl, 20 kDa) on day 9. Scale bar represents 100 μm. Arrow heads show new sprouting from pre-existing vessels, as quantified in (**b**). **P < 0.01 by ANOVA with Bonferroni’s Multiple Comparison test, *N* = 4. Asterisks indicate intussusceptive angiogenesis (not quantified). **c** Viability (left panel) and migration (right panel) of HMEC are stimulated by incubation with insulin for 72 h in medium containing reduced serum (0.5%). **P < 0.01 by ANOVA with Bonferroni’s Multiple Comparison test, *N* = 11; ^#^*P* = 0.0657 by ANOVA with Bonferroni’s Multiple Comparison test, *N* = 5. **d** Effect of insulin on endothelial sprout formation by HUVEC in vitro. After embedding of spheroids in a 3D collagen gel, the number of sprouts per spheroid (left panel) and total sprout length (right panel) were significantly increased by treatment with insulin. However, mean sprout length (middle panel) did not change. **P < 0.001 by *t*test, *N* = 6

The expression of INSR on EC suggests a role for insulin in the activation of endothelial cells. To investigate the role of insulin during angiogenesis, the in vivo chorioallantoic membrane (CAM) model was used.^37^ Topical administration of insulin on the CAM resulted in an increased number of vascular sprouts (**p < 0.01, Fig. 4a, b). Interestingly, we also observed a profound difference in the architecture of the vasculature with more tortuous vessels and an enhanced appearance of intussusceptive angiogenesis (asterisks in Fig. 4a).

Since HMEC cells had the highest expression of INSR (Supplementary Fig. S2C), we grew these cells to near-confluency and incubated them with insulin doses to verify the stimulatory effect in vitro. Cell assays for viability and migration were performed and we were able to observe significant stimulatory activities (Fig. 4c), whereas HUVEC were much less responsive to insulin in our assays (Supplementary Fig. S6A). In a separate in vitro angiogenesis assay, spheroids of EC were allowed to sprout in a 3-dimensional gel. As HMEC do not readily sprout in vitro,^28^ HUVEC were used for these assays. A significantly enhanced sprouting was observed in the presence of insulin. The enhanced in vitro sprouting was based on an increase in the number of sprouts (**p < 0.01, Fig. 4d, left panel), rather than the sprouting capacity of cells themselves (mean sprout length, Fig. 4d, middle panel). This resulted in a significant increase of the total sprout length per spheroid in the presence of insulin (**p < 0.01, Fig. 4d, right panel).

### Antagonizing insulin receptor with antibodies and siRNA counteracts the angiogenic response

**Fig. 5:**
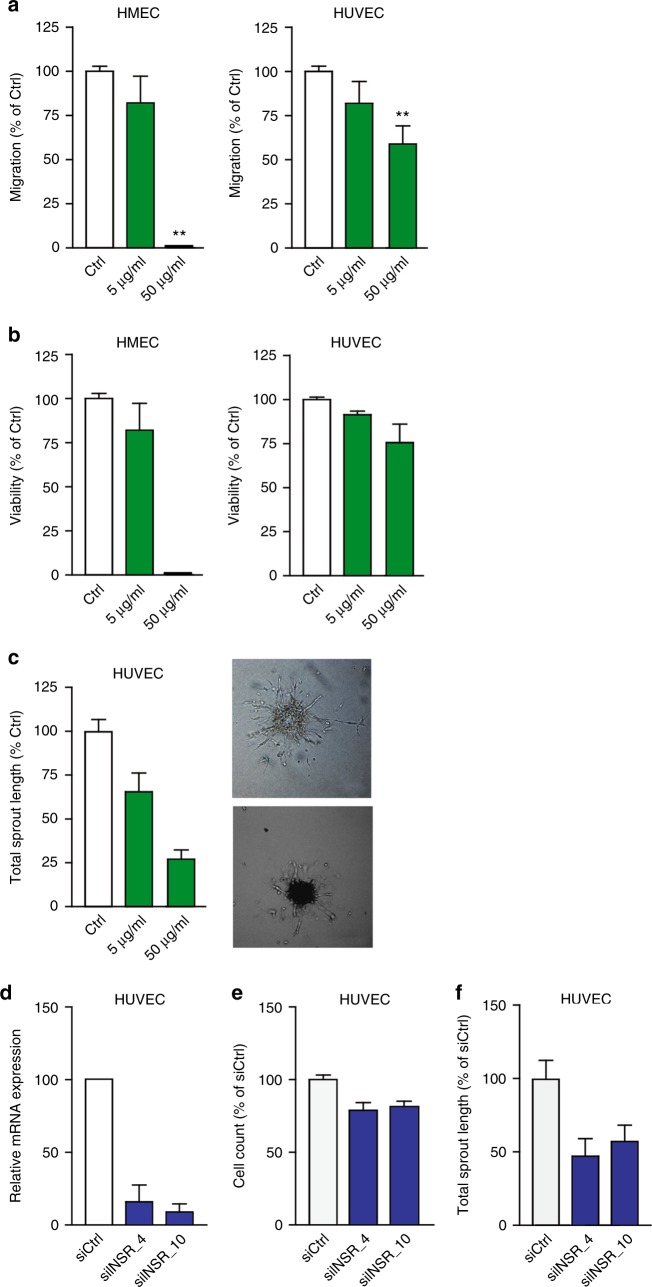
Insulin receptor antibodies inhibit EC function in vitro. **a** Inhibition of HUVEC and HMEC migration by anti-INSR polyclonal antibodies (pAb) as determined by scratch wound assay. A minor inhibitory effect is observed with low concentrations of antibody (5 μg/ml), whereas considerable inhibition is observed with high concentrations of antibody (50 μg/ml). **P < 0.01 by ANOVA with Bonferroni’s Multiple Comparison test, *N* = 2–10. **b** Inhibition of HUVEC and HMEC cell viability by treatment with anti-INSR pAb. **P < 0.01 by ANOVA with Bonferroni’s Multiple Comparison test, *N* = 14–18. **c** Antibody interference in the HUVEC sprouting assay with representative images of sprouting spheroids for Ctrl and treated with pAb at 50 μg/ml. Total sprout length (shown here), as well as mean length of the sprouts and number of sprouts per spheroid (Fig. 5) were reduced. **P < 0.01 by ANOVA with Bonferroni’s Multiple Comparison test, *N* = 7–12. **d**–**f** Effect of INSR knockdown by siRNA (**d**) on proliferation (**e**) and sprouting (**f**) of HUVEC cells was evaluated and quantified. Efficient knockdown was accomplished at the RNA level, which resulted in only a slight reduction in cell viability, but which had a pronounced impact on endothelial sprouting. Values are presented relative to control siRNA. *P < 0.05, **P < 0.01 by ANOVA with Bonferroni’s Multiple Comparison test, *N* = 4 (**d**), *N* = 9 (**e**), *N* = 11 (**f**)

To confirm the inhibitory effect of anti-INSR antibodies on EC in vitro, HUVEC and HMEC were exposed to a polyclonal anti-INSR antibody. Both migration and viability were significantly inhibited (Fig. 5a, b, respectively, **p < 0.01). Sprouting (total sprout length; Fig. 5c) was also significantly inhibited by the polyclonal antibody, which was the resultant of a reduction in the number of sprouts (**p < 0.01) and the average sprout length (**p < 0.01, Supplementary Fig. S6D). Interestingly, the monoclonal anti-INSR antibody showed a much weaker but still significant effect in the proliferation assay, while migration was unaffected by this antibody (Supplementary Fig. S6B and S6C). The prevailing effect of the polyclonal antibody over the monoclonal antibody can be the result of intrinsic differences in epitope recognition or affinity, as well as of the possibility that binding of multiple different antibodies in the polyclonal serum can more efficiently interfere with receptor function.

Independent verification of the role of INSR in the biology of EC was generated by knockdown of INSR by siRNA technology (Supplementary Material). HUVEC were transfected by two different siRNAs (siINSR_4 and siINSR_10) specific for INSR, resulting in 85–90% of mRNA suppression with both siRNAs (Fig. 5d, **p < 0.01). This significantly reduced the cell number (**p < 0.01), as compared to a scrambled siRNA (siCtrl, Fig. 5e). For the sprouting assay, siRNA transfected cells were used and grown in spheroids by hanging drop technology as described above. Both INSR siRNAs inhibited the mean sprout length and the number of sprouts per spheroid, resulting in a significantly reduced total sprout length (Fig. 5f, **p < 0.01, *p < 0.05).

**Fig. 6:**
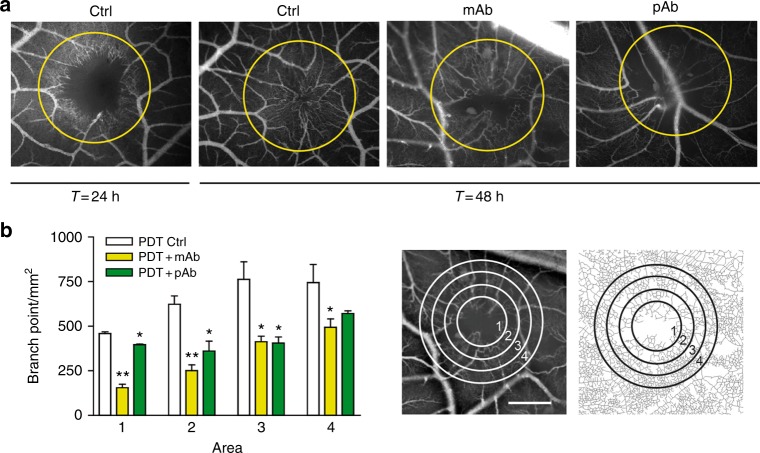
Insulin receptor antibodies inhibit angiogenesis in vivo. **a** CAMs were treated with vaso-occlusive Visudyne^®^-photodynamic therapy in the areas indicated with circles. Representative fluorescence angiographies of CAMs before (pre-PDT), 24 and 48 h after PDT are shown. Anti-INSR monoclonal antibody or a polyclonal antibody were administered topically immediately after irradiation, where indicated. The antibody treatment was repeated 24 h later. Angiogenesis in the irradiated zone was imaged 48 h after start of treatment. **b** Images were quantified using ImageJ software, and quantification using the branching points descriptor is shown. Right images show the concentric circles indicating the 4 treatment areas, and the skeletonised plot of the vasculature. Scale bar represents 200 μm. *P < *0.05*, **P < 0.01 by ANOVA with Bonferroni’s Multiple Comparison test, *N* = 3

To investigate whether intervention with the INSR pathway affects angiogenesis in vivo, an anti-INSR monoclonal and a polyclonal antibody were tested in the CAM model. Both antibodies recognise human, mouse and chicken INSR as assessed by immunohistochemistry (data not shown). In this model angiogenesis was induced by photodynamic vaso-occlusion after injection of photosensitiser Visudyne^®^ and subsequent exposure to light.^32^ At the molecular level, qPCR showed that expression of INSR was significantly induced during this angiogenic stimulation, a feature that was also visible for VEGFR2. A similar trend for VEGFR1 was observed (Supplementary Fig. S7). While under control conditions the occluded lesion was revascularized by sprouting angiogenesis over a period of 48 h (Fig. 6a), the presence of monoclonal antibody, and also but to a lesser extent of polyclonal antibody, significantly inhibited this process (Fig. 6a). Enumeration of vascular branching points/mm^2^ shows a significant suppression of angiogenesis (Fig. 6b, left panel, *p < 0.05, **p < 0.01), as quantified in four concentric areas (Fig. 6b, right panels).

## Discussion

We identified insulin receptor (INSR) as a marker of the tumour vasculature in a genomic search analysing RNA preparations from isolated colorectal carcinoma (CRC) endothelial cells (EC) and their counterparts from normal colorectal tissue samples. In this study, markers of physiologically activated endothelium were excluded by co-analysis of isolated placental endothelial cells. In an independent deep sequencing study for identification of embryo-specific genes that become re-expressed in tumour endothelial cells, INSR was also found to be a specific marker of the tumour vasculature. This overexpression of INSR suggests a function in the process of tumour angiogenesis, and points to a relationship with tumour aggressiveness and prognosis. Indeed, an inverse correlation between the expression of INSR and relapse-free survival was observed in CRC expression profiles. For these reasons we investigated the role of INSR in the biology of EC and in the formation of (tumour) vessels. This knowledge would be instrumental for the assessment of the relevance of INSR and the application of INSR targeting for angiostatic cancer therapy. Interestingly, immunohistochemical analysis of human CRC samples revealed that expression of INSR was almost exclusively found in the vasculature of colorectal tumours. Similar results were observed in ten other tumour types. We show in the current report that INSR was induced in EC by angiogenic stimulation. We present several lines of evidence for the pro-angiogenic function of INSR in EC. Firstly, INSR functions as a growth factor receptor, as exposure to insulin stimulated growth and tube formation of EC in vitro and in vivo. Secondly, targeting of INSR on activated EC displayed effective anti-angiogenic activity. Thirdly, anti-angiogenic treatment of tumours was shown to suppress the expression of vascular INSR.

Insulin receptor is part of a complex signalling axis, in which insulin-like growth factor-1 receptor (IGF1R) and IGF2R take part, together with the INSR ligands insulin and the insulin-like growth factors (IGF)-1 and -2, as well as a series of seven insulin-like growth factor-binding proteins (IGFBP1-7).^14^ Enhanced complexity comes from alternative splicing of INSR in INSR-A and -B on the one hand and heterodimerisation of INSR and IGF1R on the other.

Differences in the function of INSR-A and INSR-B now suggest that INSR targeting may have clinical impact in the field of oncology. We reviewed the background of this pathway recently.^14^ This signalling axis has pleiotropic and versatile functions in many cells and its role in EC is gradually becoming unveiled. It is important to realise that the ligands, insulin and the IGFs, although binding to their cognate receptors with the highest affinity, can all bind to the other two receptors as well, albeit with lower affinity. The stimulatory effect of insulin on sprouting of EC has been reported previously,^17,38–41^ and these observations may explain why patients with type 2 diabetes and those that are treated with daily insulin supplementation have an increased risk of developing malignant neoplasms.^18^ Our observation of the correlation between INSR expression and cancer patient survival, as shown in Fig. 1d, further underscores this. In addition, patients diagnosed with insulin resistance syndrome, also called metabolic syndrome, characterised by hyperinsulinemia and chronic inflammation, are at greater risk for malignancies.^42^ Together, these considerations suggest that an angiostatic INSR targeting approach may constitute a valid anti-cancer strategy in the clinic.

The most interesting finding of the current study was that the major isoform of INSR in the tumour vasculature is the proliferation associated isoform INSR-A and not the metabolic isoform INSR-B. Furthermore, RNA sequencing demonstrated INSR-A to be the dominant embryonic form, confirming its oncofoetal characteristics. Our study independently verifies the recent results by Roudnicky et al.,^21^ who recently described INSR-A as the main isoform in tumour endothelium and endothelial cells in vitro. We furthermore provide functional studies on the role of INSR-A in vitro and in vivo and on the regulation during angiostatic treatment of patients. INSR-A is the main isoform in foetal tissues (e.g. foetal fibroblasts, muscle, liver and kidney) and in cancer, and binds insulin-like growth factor 2 (IGF-2) with high affinity, which elicits mitogenic effects,^20^ thereby contributing not only to organism development, but also to cancerous growth.

INSR and IGF1R have been previously reported as attractive therapeutic targets in cancer.^17,43–45^ Several neutralising antibodies and small molecule receptor kinase inhibitors have been developed, such as dalotuzumab and OSI-906.^46,47^ However, therapies targeting IGF1R alone or in combination with other drugs were tested but eventually revealed contradictory results.^48–51^ Resistance to IGF1R targeting agents is believed to be mediated by upregulation of INSR in tumours, especially the oncofoetal INSR-A variant, which has a high affinity for IGF2 and as such sustains proangiogenic signalling despite inhibition of IGF1R.^20,52^ Moreover, INSR-A is less involved in metabolic activities of insulin than INSR-B.^53^ It is interesting to note here that *Gallus gallus* (chicken) expresses only one isoform of INSR, being the shorter INSR-A variant. We showed that targeting of INSR in this model is extremely effective at inhibition of angiogenesis (Fig. 5). Thus, targeting of the INSR-A variants emerges as a valid angiostatic therapeutic option in cancer. While specific targeting of INSR-A seems therefore to be an attractive opportunity, this approach is not as straightforward as anticipated. This is due to the fact that INSR-A differs from INSR-B by splicing out of exon 11, a domain of 12 amino acids located at the C-terminus of INSR alpha-subunit. Although it is not trivial to target a receptor that misses a domain, it may be possible to design a compound, e.g. an antibody that is targeted towards the neoepitope in INSR-A that is the result of the truncation. Alternatively, the design of IGF-like ligands or mimics with high or exclusive affinity for INSR-A alpha-subunit containing receptors may be pursued. It remains to be investigated whether such INSR-A specific treatment can be used to target the tumour vasculature, but it definitely presents a therapeutic opportunity.

Targeting of growth factor receptors is an obvious anti-cancer strategy and many drugs are designed towards VEGFR (bevacizumab, sunitinib), EGFR (cetuximab, erlotinib), HER2 (trastuzumab, pertuzumab) and PDGFR (imatinib, crenolanib). Hormone receptors are growth factor receptors as well, as many hormones signal to stimulate cell growth. Although insulin is known as a hormone produced in the beta-cells of the pancreas with liver-, muscle- and fat cells as its targets, it also has growth factor functions. The strong overexpression of INSR (mainly INSR-A) in the tumour vasculature and its functional role in activation signals suggests that this signalling axis may serve as a target for therapy. Relatedly, a similar selective overexpression was found for the follicle stimulating hormone (FSH) receptor. Application of targeting the FSH receptor for therapeutic purposes was also previously suggested.^54^

Additional (pre-)clinical evidence on the importance of INSR in tumour angiogenesis was provided by the demonstration that anti-angiogenic treatment reduces vascular INSR expression. Sunitinib is a tyrosine kinase inhibitor that blocks the signalling pathways of a series of growth factor receptors, among which are VEGFR1 and VEGFR2, PDGFR-b, c-KIT, and FLT-3. In our CAM and mouse models, treatment with sunitinib significantly decreased the expression levels of endothelial INSR (Fig. 3). To state clinical relevance, we have previously shown that in RCC patients angiogenesis is clearly inhibited in the primary tumour in response to sunitinib treatment and demonstrated that this treatment led to normalisation of angiogenic growth factor expression.^24^ We now show that, in analogy to VEGFRs, INSR is also suppressed by angiostatic treatment with sunitinib. The rapid rebound of angiogenesis upon halting the treatment with sunitinib before surgery, but supposedly also in the treatment rests that occur after each treatment period of 4 weeks, is clearly associated to relative induction of INSR-A vs. INSR-B expression (Fig. 3).

Likewise, the potential of targeting INSR-A has been extensively discussed. However, cross-reactivity with INSR-B, IGF-IR and hybrid receptors has always been a major concern. Consistent with this, sunitinib has numerous side effects due to its lack of tumour specificity. It is unclear how the current results move past the long recognised difficulty of cross-reactivity. Most antibodies used in IHC will not discriminate between INSR-A and INSR-B, which usually requires mRNA or similar genomic analyses, and activated (phosphorylated) IGF-IR cannot be distinguished from phosphorylated INSRs by IHC. Overall, though theoretically, targeting the INSR-A is an exciting possibility, currently tools and technologies are lacking to accomplish this. Further insight on the relative roles of these isoforms could be generated by the development of genetically engineered mouse models (GEMMs) that allow for (conditional) expression of either isoform in the endothelium. This may open avenues to develop tools to target INSR-A variant through either genetic means, or the development of targeting moieties with specific effects on (hybrid) receptors of this isoform.

Summarising, we have demonstrated that overexpression of INSR is associated with increased angiogenesis. We therefore suggest that direct targeting of endothelial INSR, supposedly most beneficial through targeting of only INSR-A, may present an interesting treatment strategy.

## Electronic supplementary material

Supplementary Material

## Availability of data and materials

All the data generated or analysed in this study are included in the manuscript or the supplementary information.

## References

[1] Folkman J (1971). Tumor angiogenesis: therapeutic implications. N. Engl. J. Med..

[2] Griffioen AW, Molema G (2000). Angiogenesis: potentials for pharmacologic intervention in the treatment of cancer, cardiovascular diseases, and chronic inflammation. Pharmacol. Rev..

[3] Hayman SR, Leung N, Grande JP, Garovic VD (2012). VEGF inhibition, hypertension, and renal toxicity. Curr. Oncol. Rep..

[4] van Beijnum JR, Nowak-Sliwinska P, Huijbers EJ, Thijssen VL, Griffioen AW (2015). The great escape; the hallmarks of resistance to anti-angiogenic therapy. Pharmacol. Rev..

[5] Huijbers EJ (2016). Role of the tumor stroma in resistance to anti-angiogenic therapy. Drug Resist. Updat..

[6] Griffioen AW (1997). CD44 is involved in tumor angiogenesis; an activation antigen on human endothelial cells. Blood.

[7] St Croix B (2000). Genes expressed in human tumor endothelium. Science.

[8] Thijssen VL (2006). Galectin-1 is essential in tumor angiogenesis and is a target for antiangiogenesis therapy. Proc. Natl. Acad. Sci. USA.

[9] van Beijnum JR (2006). Gene expression of tumor angiogenesis dissected: specific targeting of colon cancer angiogenic vasculature. Blood.

[10] van Beijnum JR (2013). Tumor angiogenesis is enforced by autocrine regulation of high-mobility group box 1. Oncogene.

[11] van der Schaft DW (2002). The designer anti-angiogenic peptide anginex targets tumor endothelial cells and inhibits tumor growth in animal models. FASEB J..

[12] Dings RP (2006). Design of nonpeptidic topomimetics of antiangiogenic proteins with antitumor activities. J. Natl. Cancer Inst..

[13] Dings RPM (2005). Antiangiogenic peptide anginex synergizes with radiation therapy to cause tumor growth inhibition and regression via endothelial cell radiosensitization. Int. J. Cancer.

[14] van Beijnum, J. R., Pieters, W., Nowak-Sliwinska, P., Griffioen, A. W. Insulin-like growth factor axis targeting in cancer and tumour angiogenesis - the missing link. *Biol*. *Rev*. *Camb*. *Philos*. *Soc*. **92**(3):1755–1768 (2017). 10.1111/brv.12306 27779364

[15] Frasca F (2008). The role of insulin receptors and IGF-I receptors in cancer and other diseases. Arch. Physiol. Biochem..

[16] Zhang H (2010). Inhibition of cancer cell proliferation and metastasis by insulin receptor downregulation. Oncogene.

[17] Rensing KL (2010). Could recombinant insulin compounds contribute to adenocarcinoma progression by stimulating local angiogenesis?. Diabetologia.

[18] Hemkens LG (2009). Risk of malignancies in patients with diabetes treated with human insulin or insulin analogues: a cohort study. Diabetologia.

[19] Jonasson JM (2009). Insulin glargine use and short-term incidence of malignancies-a population-based follow-up study in Sweden. Diabetologia.

[20] Frasca F (1999). Insulin receptor isoform A, a newly recognized, high-affinity insulin-like growth factor II receptor in fetal and cancer cells. Mol. Cell. Biol..

[21] Roudnicky Filip, Dieterich Lothar C, Poyet Cedric, Buser Lorenz, Wild Peter, Tang Dave, Camenzind Peter, Ho Chien Hsien, Otto Vivianne I, Detmar Michael (2017). High expression of insulin receptor on tumour-associated blood vessels in invasive bladder cancer predicts poor overall and progression-free survival. The Journal of Pathology.

[22] van Beijnum JR, Rousch M, Castermans K, van der Linden E, Griffioen AW (2008). Isolation of endothelial cells from fresh tissues. Nat. Protoc..

[23] Trapnell C (2010). Transcript assembly and quantification by RNA-Seq reveals unannotated transcripts and isoform switching during cell differentiation. Nat. Biotechnol..

[24] Griffioen AW (2012). Rapid angiogenesis onset after discontinuation of sunitinib treatment of renal cell carcinoma patients. Clin. Cancer Res..

[25] Hillen F (2008). Leukocyte infiltration and tumor cell plasticity are parameters of aggressiveness in primary cutaneous melanoma. Cancer Immunol., Immunother.: CII.

[26] Jorissen RN (2009). Metastasis-associated gene expression changes predict poor outcomes in patients with dukes stage b and c colorectal cancer. Clin. Cancer Res..

[27] Pagnotta SM (2013). Ensemble of gene signatures identifies novel biomarkers in colorectal cancer activated through PPARgamma and TNFalpha signaling. PLoS ONE.

[28] van Beijnum JR, van der Linden E, Griffioen AW (2008). Angiogenic profiling and comparison of immortalized endothelial cells for functional genomics. Exp. Cell Res..

[29] Reuwer AQ (2012). Functional consequences of prolactin signaling in endothelial cells: a potential link with angiogenesis in pathophysiology?. J. Cel. Mol. Med..

[30] Nowak-Sliwinska Patrycja, Alitalo Kari, Allen Elizabeth, Anisimov Andrey, Aplin Alfred C., Auerbach Robert, Augustin Hellmut G., Bates David O., van Beijnum Judy R., Bender R. Hugh F., Bergers Gabriele, Bikfalvi Andreas, Bischoff Joyce, Böck Barbara C., Brooks Peter C., Bussolino Federico, Cakir Bertan, Carmeliet Peter, Castranova Daniel, Cimpean Anca M., Cleaver Ondine, Coukos George, Davis George E., De Palma Michele, Dimberg Anna, Dings Ruud P. M., Djonov Valentin, Dudley Andrew C., Dufton Neil P., Fendt Sarah-Maria, Ferrara Napoleone, Fruttiger Marcus, Fukumura Dai, Ghesquière Bart, Gong Yan, Griffin Robert J., Harris Adrian L., Hughes Christopher C. W., Hultgren Nan W., Iruela-Arispe M. Luisa, Irving Melita, Jain Rakesh K., Kalluri Raghu, Kalucka Joanna, Kerbel Robert S., Kitajewski Jan, Klaassen Ingeborg, Kleinmann Hynda K., Koolwijk Pieter, Kuczynski Elisabeth, Kwak Brenda R., Marien Koen, Melero-Martin Juan M., Munn Lance L., Nicosia Roberto F., Noel Agnes, Nurro Jussi, Olsson Anna-Karin, Petrova Tatiana V., Pietras Kristian, Pili Roberto, Pollard Jeffrey W., Post Mark J., Quax Paul H. A., Rabinovich Gabriel A., Raica Marius, Randi Anna M., Ribatti Domenico, Ruegg Curzio, Schlingemann Reinier O., Schulte-Merker Stefan, Smith Lois E. H., Song Jonathan W., Stacker Steven A., Stalin Jimmy, Stratman Amber N., Van de Velde Maureen, van Hinsbergh Victor W. M., Vermeulen Peter B., Waltenberger Johannes, Weinstein Brant M., Xin Hong, Yetkin-Arik Bahar, Yla-Herttuala Seppo, Yoder Mervin C., Griffioen Arjan W. (2018). Consensus guidelines for the use and interpretation of angiogenesis assays. Angiogenesis.

[31] Nowak-Sliwinska P (2011). Organometallic ruthenium(ii) arene compounds with antiangiogenic activity. J. Med. Chem..

[32] Nowak-Sliwinska P, van Beijnum JR, van Berkel M, van den Bergh H, Griffioen AW (2010). Vascular regrowth following photodynamic therapy in the chicken embryo chorioallantoic membrane. Angiogenesis.

[33] Lim SH (2010). The neovessel occlusion efficacy of 15-hydroxypurpurin-7-lactone dimethyl ester induced with photodynamic therapy. Photochem. Photobiol..

[34] Gotink KJ (2014). Acquired tumor cell resistance to sunitinib causes resistance in a HT-29 human colon cancer xenograft mouse model without affecting sunitinib biodistribution or the tumor microvasculature. Oncoscience.

[35] Thijssen VL, Brandwijk RJ, Dings RP, Griffioen AW (2004). Angiogenesis gene expression profiling in xenograft models to study cellular interactions. Exp. Cell Res..

[36] Gan HK, Seruga B, Knox JJ (2009). Sunitinib in solid tumors. Expert. Opin. Investig. Drugs.

[37] Nowak-Sliwinska P, Segura T, Iruela-Arispe ML (2014). The chicken chorioallantoic membrane model in biology, medicine and bioengineering. Angiogenesis.

[38] Liu Y, Petreaca M, Martins-Green M (2009). Cell and molecular mechanisms of insulin-induced angiogenesis. J. Cell. Mol. Med..

[39] Zeng G, Quon MJ (1996). Insulin-stimulated production of nitric oxide is inhibited by wortmannin. Direct measurement in vascular endothelial cells. J. Clin. Invest..

[40] Michell BJ (1999). The Akt kinase signals directly to endothelial nitric oxide synthase. Curr. Biol.: CB.

[41] Bach LA (2015). Endothelial cells and the IGF system. J. Mol. Endocrinol..

[42] Giovannucci E (2007). Metabolic syndrome, hyperinsulinemia, and colon cancer: a review. Am. J. Clin. Nutr..

[43] Belfiore A, Frasca F, Pandini G, Sciacca L, Vigneri R (2009). Insulin receptor isoforms and insulin receptor/insulin-like growth factor receptor hybrids in physiology and disease. Endocr. Rev..

[44] Frasca F, Pandini G, Vigneri R, Goldfine ID (2003). Insulin and hybrid insulin/IGF receptors are major regulators of breast cancer cells. Breast Dis..

[45] Heidegger I, Kern J, Ofer P, Klocker H, Massoner P (2014). Oncogenic functions of IGF1R and INSR in prostate cancer include enhanced tumor growth, cell migration and angiogenesis. Oncotarget.

[46] Singh P, Alex JM, Bast F (2014). Insulin receptor (IR) and insulin-like growth factor receptor 1 (IGF-1R) signaling systems: novel treatment strategies for cancer. Med. Oncol..

[47] Arcaro A (2013). Targeting the insulin-like growth factor-1 receptor in human cancer. Front. Pharmacol..

[48] Di Cosimo, S. et al. Combination of the mTOR inhibitor ridaforolimus and the anti-IGF1R monoclonal antibody dalotuzumab: preclinical characterization and phase i clinical trial. *Clin. Cancer Res.* **21**(1):49–59 (2015). 10.1158/1078-0432.CCR-14-0940 PMC5705197 25320355

[49] Puzanov I., Lindsay C. R., Goff L., Sosman J., Gilbert J., Berlin J., Poondru S., Simantov R., Gedrich R., Stephens A., Chan E., Evans T. R. J. (2014). A Phase I Study of Continuous Oral Dosing of OSI-906, a Dual Inhibitor of Insulin-Like Growth Factor-1 and Insulin Receptors, in Patients with Advanced Solid Tumors. Clinical Cancer Research.

[50] Reidy DL (2010). Randomized, phase II study of the insulin-like growth factor-1 receptor inhibitor IMC-A12, with or without cetuximab, in patients with cetuximab- or panitumumab-refractory metastatic colorectal cancer. J. Clin. Oncol..

[51] Moran T (2014). Activity of dalotuzumab, a selective anti-IGF1R antibody, in combination with erlotinib in unselected patients with Non-small-cell lung cancer: a phase I/II randomized trial. Exp. Hematol. & Oncol..

[52] Bid HK, Zhan J, Phelps DA, Kurmasheva RT, Houghton PJ (2012). Potent inhibition of angiogenesis by the IGF-1 receptor-targeting antibody SCH717454 is reversed by IGF-2. Mol. Cancer Ther..

[53] Sciacca L (2003). Signaling differences from the A and B isoforms of the insulin receptor (IR) in 32D cells in the presence or absence of IR substrate-1. Endocrinology.

[54] Radu A (2010). Expression of follicle-stimulating hormone receptor in tumor blood vessels. N. Engl. J. Med..

[55] Mesri M (2013). Identification and characterization of angiogenesis targets through proteomic profiling of endothelial cells in human cancer tissues. PLoS ONE.

