# Supplementary material for: Oncofoetal insulin receptor isoform A marks the tumour endothelium; an underestimated pathway during tumour angiogenesis and angiostatic treatment
Source: Br J Cancer. 2018 Dec 18;120(2):218–28. doi: 10.1038/s41416-018-0347-8 (PMC6342959; doi:10.1038/s41416-018-0347-8)
Supplement: Supplementary file 1 — Supplementary Material [file 41416_2018_347_MOESM1_ESM.docx]

**Supplementary Material_Nowak-Sliwinska et al.**

1. **Supplementary Figures**
2. **Supplementary Table**
3. **Supplementary Methods**
4. **Supplementary Figures**

**
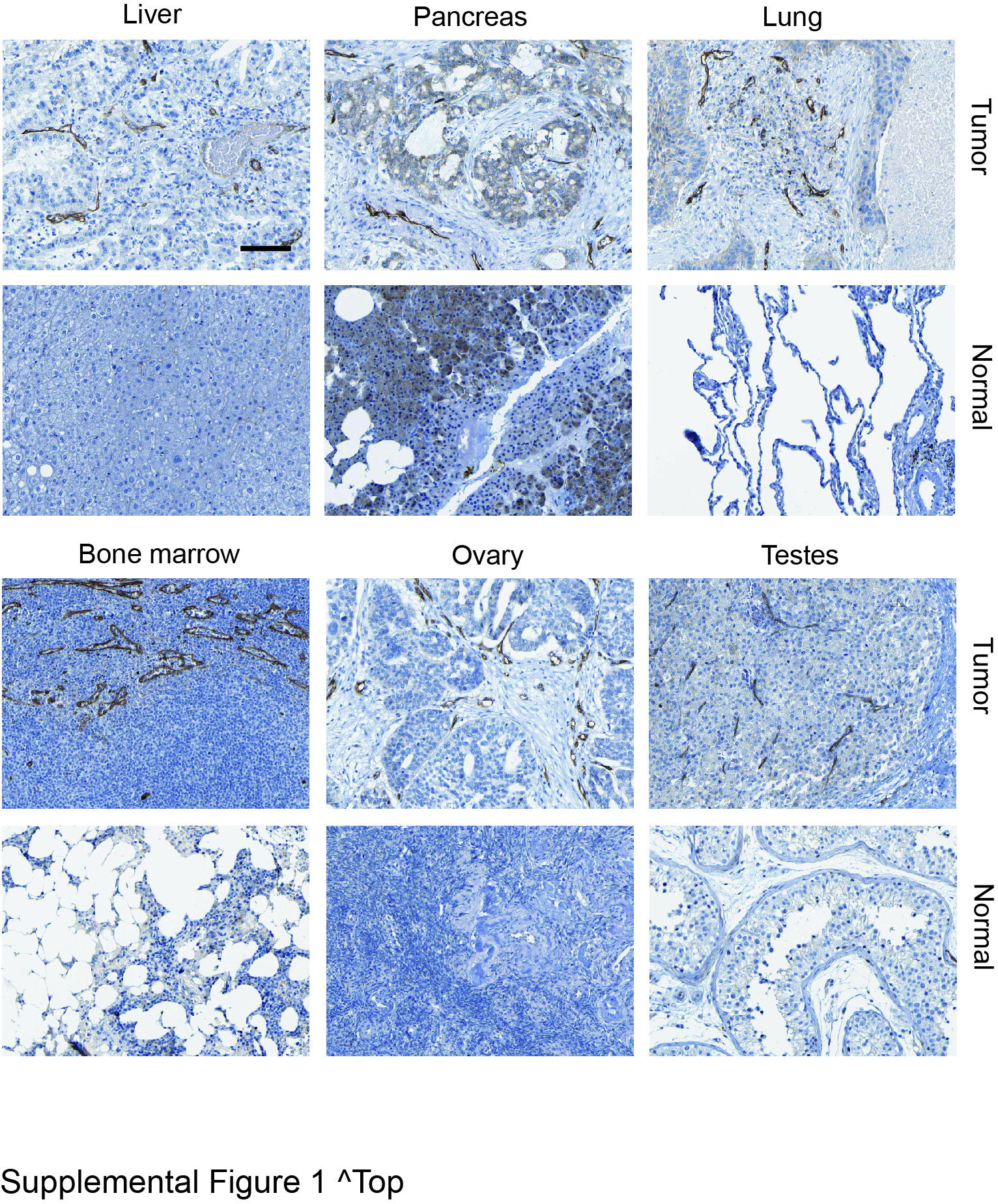
**

**Supplementary Figure S1:** Vascular overexpression of INSR in different tumor types. Immunohistochemical staining of a panel of human tumors and corresponding normal tissues indicates pronounced vascular overexpression of INSR. Scale bar = 100 μm.


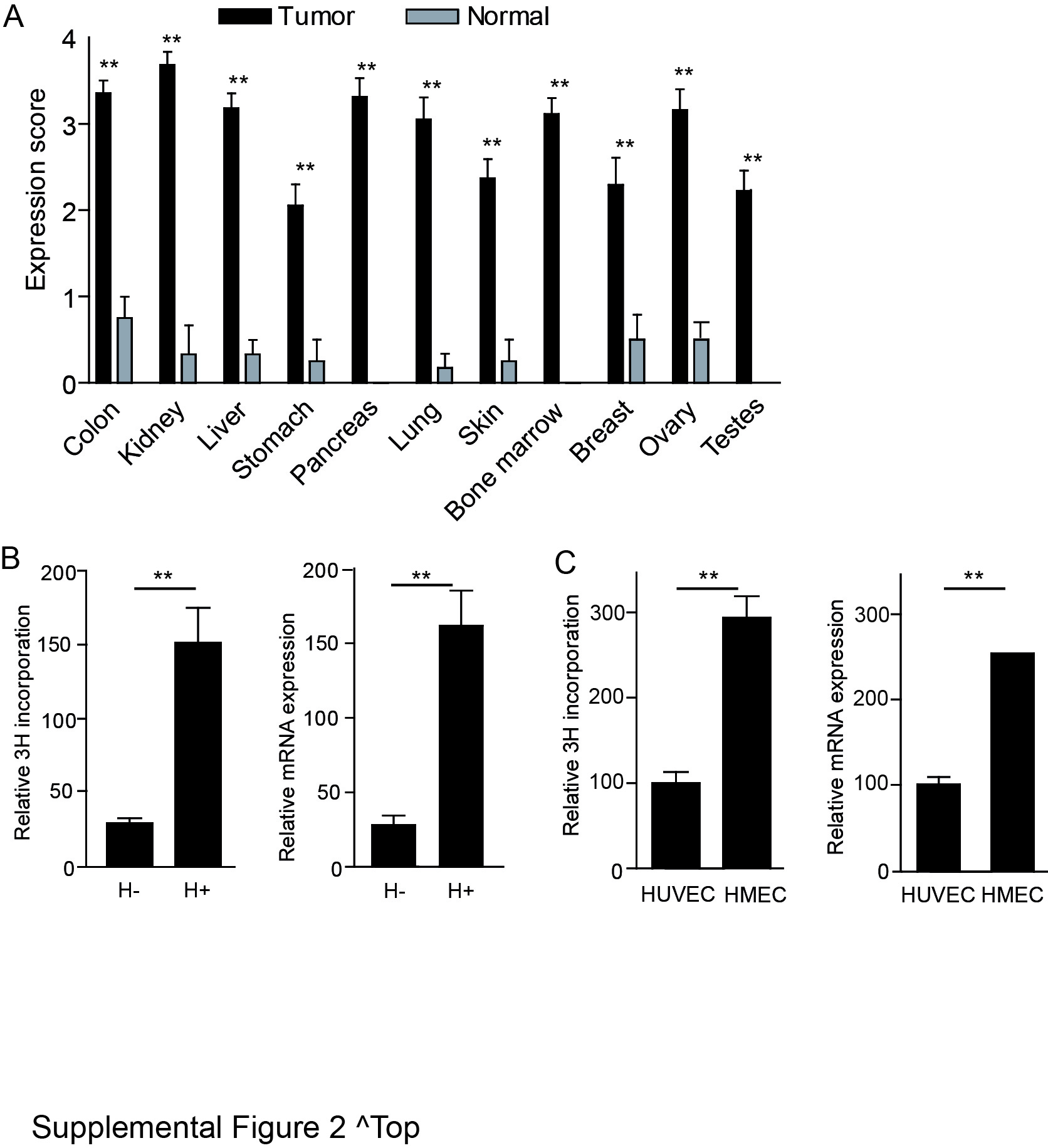


**Supplementary Figure S2:** Overexpression of INSR in tumor endothelium and activated endothelial cells. **(**A) Quantification of vascular INSR expression in different tumor types.

Quantification of the INSR expression score in 11 tumor types (source: Human Protein Atlas). The exact numbers of analyzed tumor samples are following: liver (n=22), colon (n=20), kidney (n=19), stomach (n=18), pancreas (n=16), lung (n=18), skin (n=19), bone marrow (n=19), breast (n=17), ovary (n=18) and testes (n=18). Tissues were scored according to the scale values: 0 - no expression; 1 - light staining; 2 - medium staining; 3 - strong staining; 4 - very strong staining. ***P<*0.01 by *t-*test. (B, C) Proliferative status measured by ^3^H-thymidine incorporation assay and INSR expression measured by qPCR in growth factor stimulated HUVEC (H+) as compared to starved HUVEC (H-) (B), and in HUVEC as compared to HMEC (C). ***P<*0.01 by *t-*test, N=2-6.


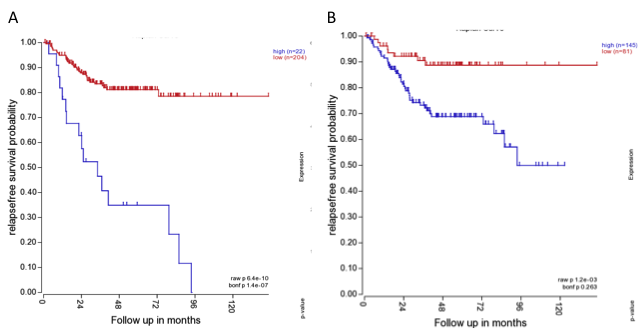


**Supplementary Figure S3:** Kaplan-Meier survival analysis of 226 CRC patients based on average (A) FLT1 and KDR (B) expression.


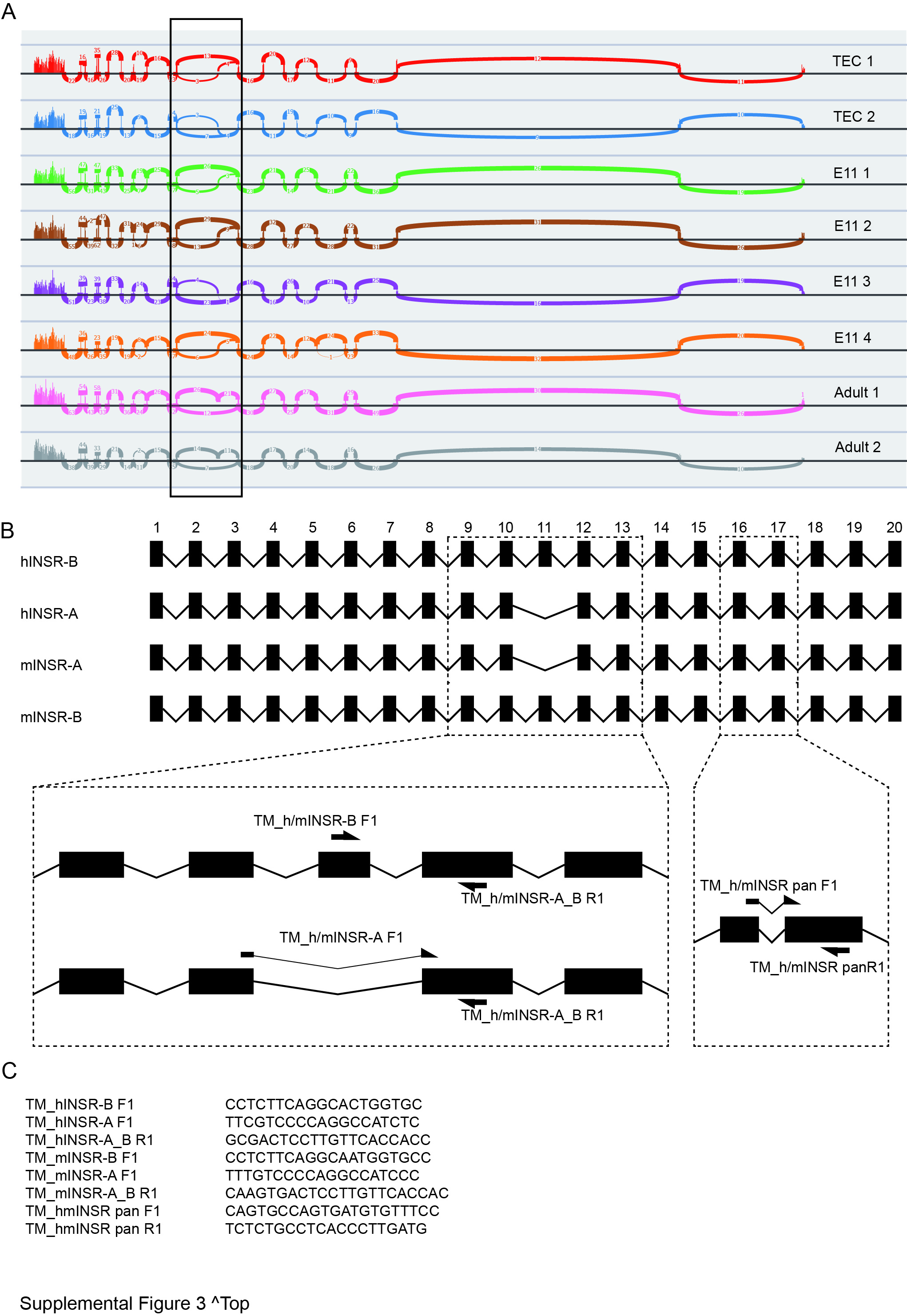


**Supplementary Figure S4:** INSR isoform expression and detection.

(A) Sashimi plots of RNA seq data on isolated tumor EC (TEC 1&2), 11-day-old mouse embryos (E11 1-4) and adult mouse (Adult 1&2). Counts on intron-spanning reads are represented as scaled arches. The area surrounding exon 11 is boxed and it is apparent that in TEC and E11 samples the exon skipping isoform A (INSR-A) is dominantly expressed over INSR-B, in contrast to Adult mouse where INSR-B is more dominant. (B) Schematic overview of mouse and human INSR and positioning of variant-specific primers. (C) Primer sequences used to detect variant-specific transcripts.


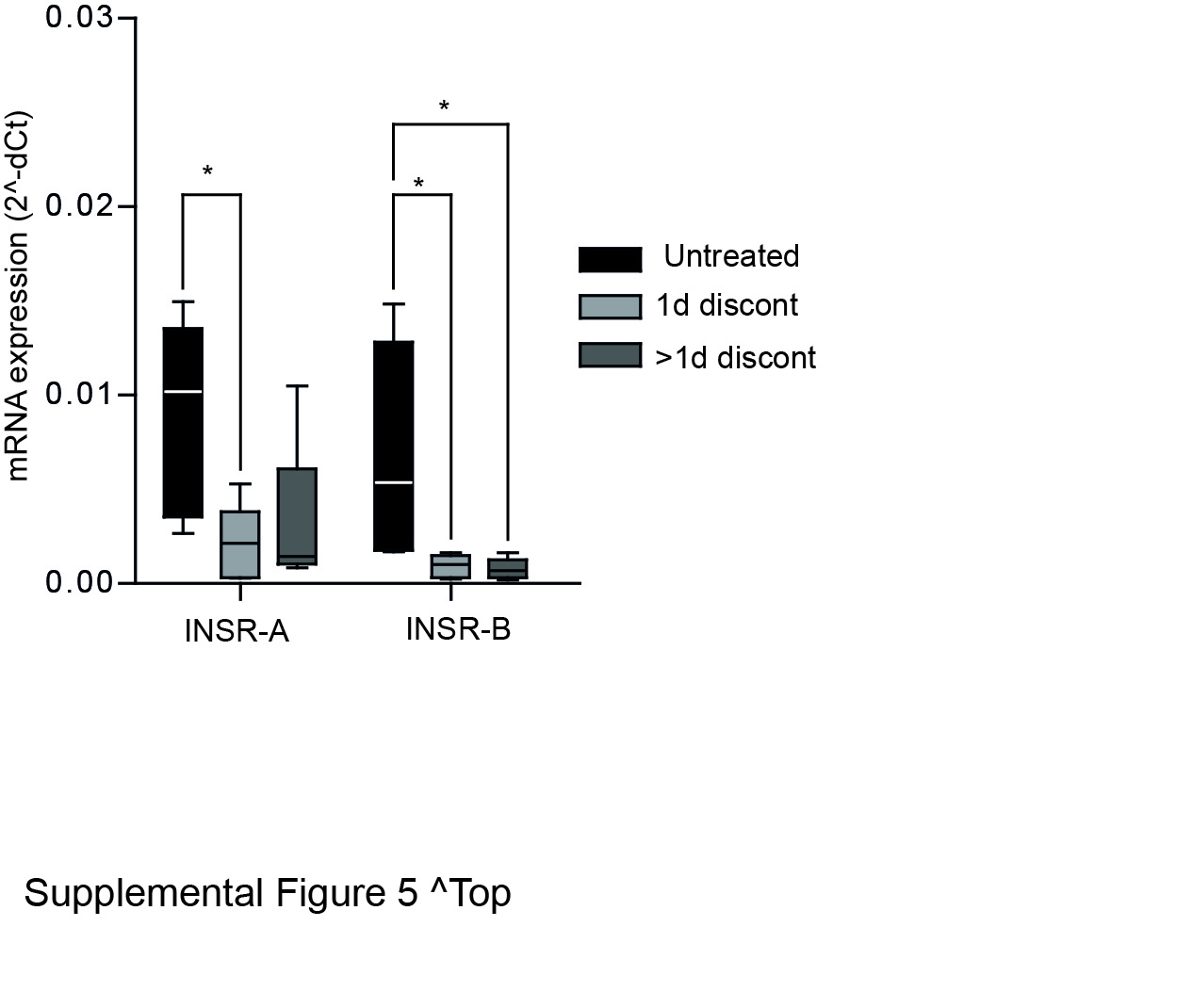


**Supplementary Figure S5:** Expression of total INSR and INSR-B in RCC tissues.

Patients received no treatment or were treated with sunitinib and surgery was performed either 1 day or more than 1 day after halting the sunitinib treatment. **P*<0.05, ***P*<0.01 by ANOVA, with Bonferroni correction, N=5-6.

**
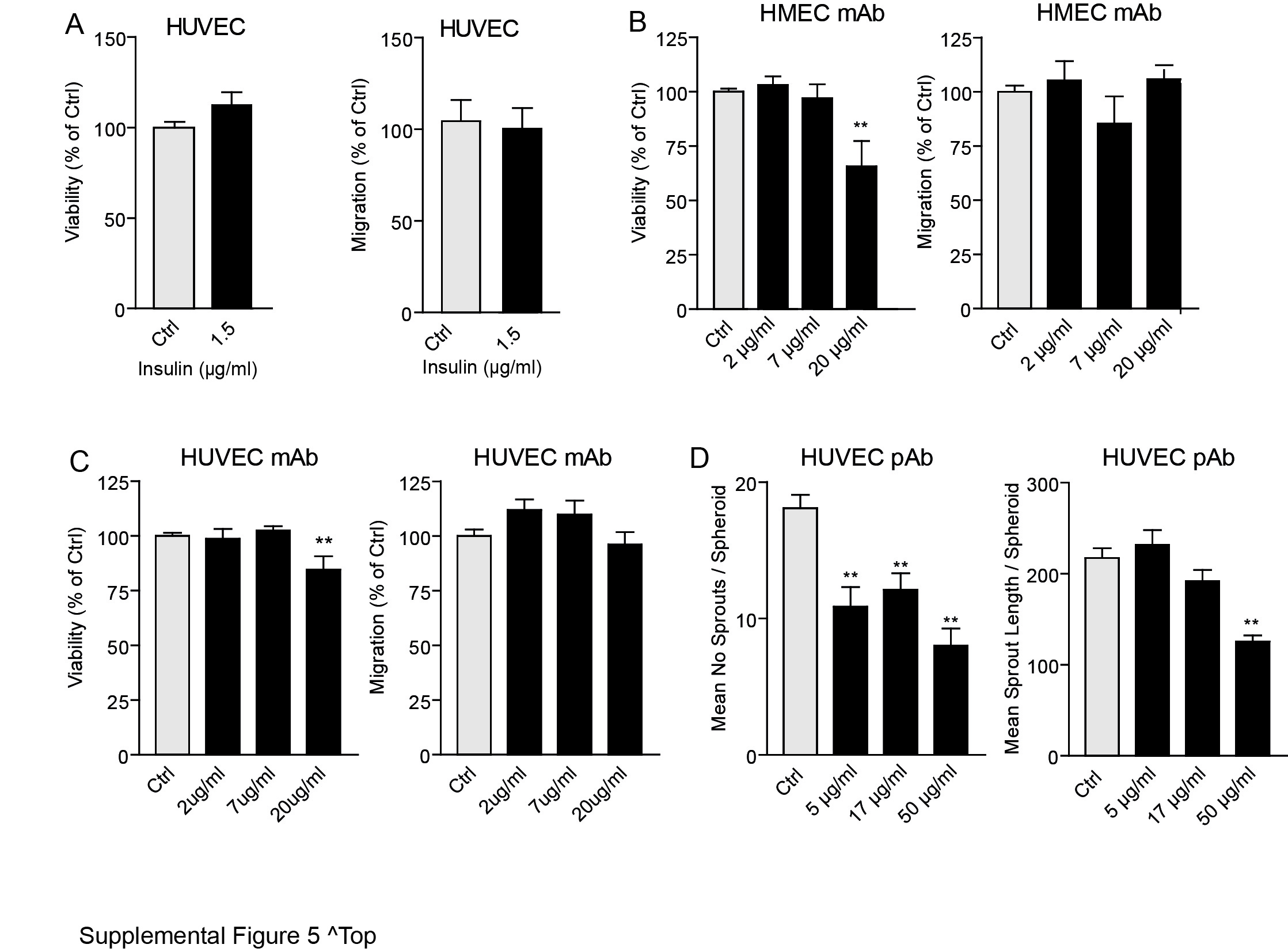
**

**Supplementary Figure S6:** Effects of insulin and INSR antibodies on angiogenesis.

(A) Insulin has no effect on viability (left panel) and migration (right panel) of HUVEC. (B,C) INSR monoclonal antibody (mAb) has minor inhibitory actions on HMEC (B, left panel) and HUVEC (C, left panel) but no effect on migration (B, C right panels). ** *P<*0.01 by ANOVA with Bonferroni correction, N=10-26. (D) Extension on sprouting parameters shown in figure 5C. Number of sprouts per spheroid and mean sprout length are decreased after incorporation of INSR polyclonal antibodies in the 3D matrix. ** *P<*0.01 by ANOVA with Bonferroni correction, N=7-12.


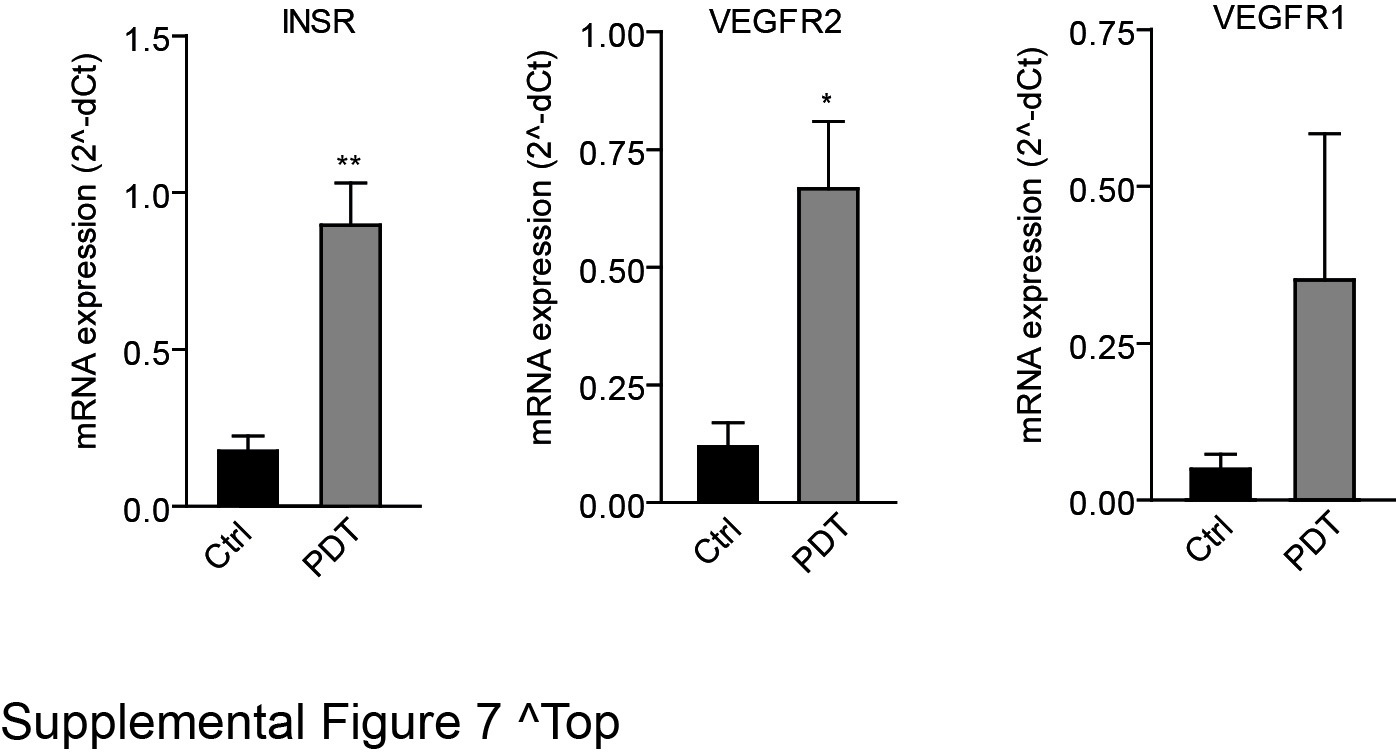


**Supplementary Figure S7:** Induction of angiogenic growth factor receptors after PDT in the CAM.

Untreated and Visudyne^®^-PDT treated CAMs analyzed for expression of INSR, VEGFR1 and VEGFR2 by qPCR. A clear induction of these receptors is evident after PDT. **P<0.05,* ***P*<0.01 by *t-*test, N=5.

**Supplementary Table**

**Supplementary Table S1.** PCR primers used in this study.

| **Human** | Fw | Rev |
| --- | --- | --- |
| INSR | TTCTCAAGGGTGCGAGCTCATC | TCCTCCCTTGGCCACCAATG |
|  |  |  |
| VEGFR1 | GCAAGGATGCATTCTTAAGC | TCAGCAGCTCAAGTGTCACC |
| VEGFR2 | GAAACCTGTCCACTTACCTGA | TTTCAGATCCACAGGGATTG |
|  |  |  |
| ACTB | GCTGTGCTACGTCGCCCTG | GGAGGAGCTGGAAGCAGCC |
| PPIA | CTCGAATAAGTTTGACTTGTGTTT | CTAGGCATGGGAGGGAACA |
| B2M | TCCATCCGACATTGAAGTTG | CGGCAGGCATACTCATCTT |
|  |  |  |
|  |  |  |
|  |  |  |
| **Chicken** | Fw | Rev |
| INSR | AATGCACTCAGGAATAACGC | CGTTGGGGAGCAGAGGC |
|  |  |  |
| VEGFR1 | TCGACACTATCTTCACAGCGG | GCTTCTGCAGTTTGGGCT |
| VEGFR2 | TCACGCCTTACAGACACCCT | AGGGAGATGTTACGGAGAATG |
|  |  |  |
| ACTB | AGACAGCTACGTTGGTGATGAA | TGCTCCTCAGGGGCTACTCT |
| PPIA | AAGGAGGGGATGAACGTG | AGCTGCCCGCAGTTGGA |
| B2M | ACCCACCCAGGATCACCA | TGTAGACGGCTTCGCTGC |
|  |  |  |
|  |  |  |
|  |  |  |
| **Mouse** | Fw | Rev |
| INSR | TCTCACCACCAAGAACTCGTG | ATGCACGCAGGAAAGAACC |
|  |  |  |
| VEGFR1 | TCAGCAGCTCAAGTGTCACC | GCTGCTTGGAGATCTCACTG |
| VEGFR2 | TTGGAAACCTATCAACTTACTTAC | TTTCAGATCCACGGAGAGCT |
|  |  |  |
| ACTB | GAAGCTGTGCTATGTTGCTCTA | GGAGGAAGAGGATGCGGCA |
| PPIA | ATTTCTTTTGACTTGCGGGC | AGCTAGACTTGAAGGGGAATG |
| B2M | CCGCCTCACATTGAAATCC | CTCTGCAGGCGTATGTATCAG |

**Supplementary Methods**

*Cells and reagents*

Primary HUVECs were routinely isolated from umbilical cords and cultured in RPMI supplemented with 10% fetal calf serum, 10% human serum and 1% penicillin/streptomycin, and grown in 1% gelatin-coated tissue culture flasks. Endothelial cell line HMEC-1 (further referred to as HMEC) was cultured in RPMI supplemented with 10% fetal calf serum. Activation of HUVEC was performed by culturing in medium supplemented with VEGF (10 ng/ml, Sigma-Aldrich, St Louis, MO) and bFGF (20 ng/ml, Reliatech, Wolfenbüttel, Germany), whereas silencing entailed culturing the cells in RPMI with 2% human serum only. HT29 cells originated from the American Tissue Culture Collection (ATCC) and were authenticated by STR profiling (Baseclear, Leiden, Netherlands). Antibodies (monoclonal anti-insulin receptor alpha-subunit, clone SPM258, AnaSpec, Inc. San Jose, CA and polyclonal anti-insulin receptor alpha-subunit,antibodies-Online, ABIN343394) were dialyzed to PBS or 0.9% NaCl (for CAM) to remove traces of azide for use in *in vitro* bioassays or *in vivo* treatment. Insulin (Actrapid Penfill, 100 IE/ml) was purchased from NovoDordisk B.V. (Bagsvaerd, Denmark). Sunitinib malate (Pfizer Global Pharmaceuticals) was dissolved at a concentration of 8 mg/ ml in a vehicle containing distilled water with 1.8% NaCl, 0.5% carboxymethylcellulose, 0.4% Tween 80 and 0.9% benzylalcohol (pH adjusted to 6.0).

siRNAs against human INSR (Qiagen; Hs_INSR_4 FlexiTube siRNA (SI00004522) and Hs_INSR_10 FlexiTube siRNA (SI05112366)) and negative control scrambled siRNA (Eurogentec) were transfected using HiPerfect transfection reagent (Qiagen). Briefly, 5 pmol siRNA was combined with 1.5 μl HiPerfect in 25 μl Optimem and allowed to complex in the gelatin-coated well for 15 minutes. Subsequently, cells (1.5*10^4^) were seeded on top and used for phenotyping after 48 hours. Although specific knockdown of either variant through siRNA or antisense oligonucleotides (for analysis of phenotypic / angiogenic contribution) would seem a tangible approach, due to sequence and design constraints, this proved not feasible with conventional approaches.

*Endothelial sprouting assay*.

EC spheroids were created using the hanging drop method [^1^](#_ENREF_1). EC were suspended in medium containing 20% methocel (Sigma-Aldrich) at 4x10^4^ cells/ml, and 25 μl drops (containing 1000 cells) were aliquoted on the inside of the lid of a Petri dish. The lid was subsequently inverted to create the hanging drops and placed over the PBS containing Petri dish. After 24 h the spheroids were harvested and embedded in a collagen gel (2 mg/mL) at 20 spheroids per well of a 96-well plate. After solidification of the gel, the medium containing the test compounds (insulin, anti-IR antibodies) was added and cells were allowed to sprout into the collagen for 16 h. In siRNA knock-down experiments, ECs were transfected with the indicated siRNA prior to the hanging drop procedure. Sprouting spheroids were pictured directly under a microscope (Leica Microsystems GmbH, Wetzlar, Germany) under 10x magnification. Quantification of sprouting was performed using a semi-automatic Image-J-based macro [^1^](#_ENREF_1).

*Isolation of endothelial cells and tissue processing for transcriptome analysis*

All mouse experiments were approved by the local Animal Ethics Committee of the VU University (reg. no. AngL13-01 and AngL13-02). Murine tumors were minced, collagenase digested and EC were isolated using magnetic beads (Life Technologies, Bleiswijk, The Netherlands). Single cell suspensions were stained with anti-mouse CD31 antibody PE-labeled (BD Pharmingen), anti-mouse CD34 antibody PE labeled (BD Pharmingen) and the pan-leukocyte marker CD45 (anti-mouse CD45 antibody APC labeled, BD Pharmingen) to be able to separate the EC population from CD31/CD45 positive macrophages. Subsequently, CD31^+^/CD34^+^ endothelial cells were separated by fluorescent activated cell sorting (BD FACSAria). Sorted ECs were immediately resuspended in Trizol (Life Technologies) and RNA was isolated according to the TRIzol reagent protocol (Life Technologies). Small amounts of RNA were co-precipitated with glycogen (Calbiochem) to increase RNA yield. The RNA quality was determined with a Bioanalyzer (Agilent Technologies).

For comparative whole transcriptome analysis, 4 embryos per time point (embryonic day) were processed. Adult mice were homogenized mechanically with a hammer in a stainless steel lid filled with liquid nitrogen. Small pieces were immediately put into Trizol, in total ca. 100 ml per mouse. Embryos and adult mouse samples were homogenized (homogenizer, handled rotor-stator homogenizer, TissueRuptor (Qiagen)) to optimize RNA isolation.

*Chorioallantoic membrane of the chicken embryo (CAM) assay*

Fertilized chicken White Leghorn eggs were incubated in a hatching incubator with a relative air humidity of 65% and a t*e*mperature of 37°C. CAMs were treated from incubation day 7 to 8 with insulin at the indicated concentrations. Vasculature was visualized on day 9 by injection of fluorescein isothiocyanate-conjugated dextran (FITC-dextran, 20 kDa, 25 mg/ml, Sigma-Aldrich) as previously described [^2^](#_ENREF_2). Fluorescence images were acquired with an F-view II 12-bit monochrome Peltier-cooled digital CCD camera driven with analysis DOCU software from Soft Imaging System, Münster, Germany. Vascular morphology was analyzed by counting vascular sprouts in fluorescence angiographies at 4x magnification.

*Photodynamic therapy (PDT) of the CAM*

Photodynamic therapy (PDT) of the CAM was performed using intravenously administrated photosensitizer Visudyne^®^ (0.2 mg of verteporfin/kg, liposomal formulation; Novartis Ophthalmics, Hettlingen, Switzerland). At 1 min after Visudyne^®^ injection, the CAM was irradiated with a light dose of 5 J/cm^2^ and irradiance of 35 mW/cm^2^ (λ_ex_ = 420 ± 20 nm, Nikon, Japan). Within PDT-treated areas, 20 μl anti-INSR monoclonal or polyclonal antibodies (10 μg/ml) were administered topically twice, immediately after light exposure and 24 h later.

## *Image acquisition and vessel quantification on the CAM*

Visualization of the CAM vasculature and irradiation with light during PDT was performed under an epi-fluorescence microscope (Nikon AG, Eclipse E 600 FN, Japan) with objectives (Plan Apo 4x/0.2, working distance: 20 mm or Plan Fluor 10x/0.3, working distance: 16 mm; Nikon AG, Japan), as previously described [^2^](#_ENREF_2). Shortly, PDT was performed (λ_ex_ = 420 ± 20 nm, λ_em_ ≥ 470 nm, Nikon, Japan) using Visudyne^®^ (Novartis Pharma Inc., Hettlingen, Switzerland). Visualization of blood vessels was achieved via fluorescence angiography obtained after intravenous (i.v.) injection of fluorescein isothiocyanate dextran (FITC-dextran, 20 kDA, 20 μl, 25 mg/ml, Sigma-Aldrich). 20 μl of India ink was administered under the CAM to enhance vascular contrast. Fluorescence images were taken using an F-view II 12-bit monochrome Peltier-cooled digital CCD camera. Image-processing and quantification of the fluorescence angiographies was achieved using a macro written in ImageJ (version 1.40 a). The four concentric circles with “1” being the central area, and “4” being the most peripheral area, create four zones of revascularization, each of which is analyzed separately by the software [^3^](#_ENREF_3). Branching points/mm^2^ was used as a descriptor of vascular morphology.

## *PDT and sunitinib combination therapy on the CAM*

Sunitinib was purchased from Genentech (San Francisco, USA), sunitinib from Pfizer Inc. (New York, USA). And was administered intravenously (20 μl) on EDD 10 and 11 at concentration: sunitinib (71 μg/kg). Concentrations were calculated for an estimated embryo weight of 3 g [^4^](#_ENREF_4). Angiograms of the CAM were taken on EDD 12. Visudyne*^®^*-PDT was performed as described above. The irradiation area was limited to a circular spot of 0.02 cm^2^ using an optical diaphragm. Directly after PDT, 20 μl of sunitinib was administered intravenously Treatment was repeated 24 hours after PDT. 48 h post PDT the CAMs were fixed prior qPCR procedure.

**References**

1. Nowak-Sliwinska P, van Beijnum JR, Casini A, Nazarov AA, Wagnières G, van den Bergh H *et al.* Organometallic Ruthenium(II) Arene Compounds with Antiangiogenic Activity. *Journal of Medicinal Chemistry* 2011; **54**(11): 3895-3902; doi 10.1021/jm2002074.

2. Reuwer AQ, Nowak-Sliwinska P, Mans LA, van der Loos CM, von der Thüsen JH, Twickler MT *et al.* Functional consequences of prolactin signaling in endothelial cells: a potential link with angiogenesis in pathophysiology? *Journal of Celular and Molecular Medicine* 2012; **16**(9): 2035-2048.

3. Nowak-Sliwinska P, Weiss A, Beijnum JR, Wong TJ, Ballini JP, Lovisa B *et al.* Angiostatic kinase inhibitors to sustain photodynamic angio-occlusion. *Journal of cellular and molecular medicine* 2012; **16**(7): 1553-1562; e-pub ahead of print 2011/09/02; doi 10.1111/j.1582-4934.2011.01440.x.

4. Romanoff AL. *The Avian Embryo: structural and functional development*. McMillan: New York, 1960.
